# Longitudinal analysis of pupillometry according to type of pharmacotherapy in Parkinson’s disease patients

**DOI:** 10.1038/s41598-025-15306-z

**Published:** 2025-08-20

**Authors:** Olga Bartošová, Petr Dušek, Martin Šíma, Daniel Stránský, Jan Hlaváč, Evžen Růžička, Karel Šonka, Ondřej Slanař

**Affiliations:** 1 Present Address: Institute of Pharmacology, First Faculty of Medicine, Charles University and General University Hospital in Prague, Prague, Czech Republic; 2 Present Address: Department of Neurology, Center of Clinical Neuroscience, First Faculty of Medicine, Charles University and General University Hospital, Prague, 121 08 Czech Republic

**Keywords:** Pupillometry, L-DOPA, Ropinirol, Parkinson’s disease, Equivalent dose, Biomarkers, Diseases, Neurology

## Abstract

The aim of this study was to evaluate the effect of different types of pharmacotherapy on pupillometric parameters in PD patients. Dopamine was shown to induce mydriasis by excitation of alpha-adrenergic receptors at the dilator pupillae muscle. Pupil diameter may thus serve as an indirect measure of peripheral dopaminergic activity. A total of 63 PD patients were treated with different types of dopaminergic pharmacotherapy. Pupil examination was performed before the first application of dopaminergic pharmacotherapy and then after one year of treatment in the OFF state and in the ON state. There were significant differences in pupillometric parameters obtained at baseline and at year 1 OFF and ON. The pupillometry did not differ among type of used pharmacotherapy in most pupillometric parameters. Two-way ANOVA confirmed the significant effect of visit in the pupillometric parameters MAX and MIN, but did not confirm the significant effect treatment between groups of patients with L-DOPA pharmacotherapy and with dopamine agonist. Linear regression analysis showed significant dose-response relationships between the most pupillometric parameters and L-DOPA equivalent doses in the patients treated with L-DOPA and dopamine agonist. In conclusion, this analysis does not confirm a differential pupillary response according to type of pharmacotherapy in PD patients.

**Supplementary Information:**

The online version contains supplementary material available at 10.1038/s41598-025-15306-z.

## Background

Parkinson’s disease (PD) is the second most common neurodegenerative disorder. It affects about 1% of people over 60, rising to 3% among those 80 and older, with men more commonly affected than women^1^. The likely cause of PD is abnormal aggregation of alpha-synuclein in the nervous system, which ultimately affects dopaminergic neurons in the substantia nigra, resulting in dopamine deficiency^2^. Although treatment of PD has become complex accounting for various non-motor symptoms, its efficacy is still mainly pronounced in the improvement of motor behavior with L-DOPA as the main symptomatic drug^3^. The use of L-DOPA is associated with a number of potential side effects, most notably long-term motor complications^4^. They mainly result from progressive degeneration of dopaminergic neurons together with fluctuations of L-DOPA concentrations in the plasma due to its short peripheral half-life^5^. Other oral therapies, like monoamine oxidase-B inhibitors (MAO-B-I), dopamine agonists, the N-methyl-D-aspartate antagonist and dopamine reuptake blocker amantadine or catechol-O-methyltransferase inhibitors (COMT-I) may be additionally utilized^6^.

It has been previously suggested that L-DOPA and dopamine exert alpha-adrenergic effects at the periphery^7–9^. Circulating dopamine may displace noradrenaline from the peripheral adrenergic nerve endings, including those innervating pupillary muscles^10^. Mydriasis induced by excitation of alpha-adrenergic receptors at the muscle dilator pupillae can be considered as an indicator of dopamine availability at the periphery^7,8^.The sympathetic pathway begins in the hypothalamus and descends to the ciliospinal center in the spinal cord (at C8-Th2 level), where it switches to preganglionic neurons. These further project to the superior cervical ganglion and from there to the ciliary body and into the m. dilatator pupillae. The sympathetic pathway is further influenced by cortical and subcortical regions including frontal cortex, limbic system, hippocampus, amygdala, and thalamus. M. constrictor pupillae and m. ciliaris are innervated by the parasympathetic system. The pathway originates from the Edinger–Westphal nucleus, continues through n. oculomotorius and switches to ciliary ganglion, from where are extraocular muscle fibers directly innervated^11^.

Pupil size and reactivity can be quantitatively assessed by pupillometry which provides important information about functioning of the autonomic nervous system^12^.

The pupillary light reflex may be affected by neurodegenerative changes in the autonomic nervous system, and pupillometry has previously been studied as a potential biomarker of autonomic dysfunction in both prodromal and manifest PD^13^. However, measuring pupillometric parameters is also a sensitive, non-invasive method for evaluating the dopaminergic activity of various pharmacotherapies commonly used in PD patients^14^. Since plasma levels of L-DOPA and dopamine agonists are not routinely available, pupillometry has been proposed as a tool to assess the plasma levels of dopaminergic treatment^12^. Yet, it is unclear how pupillometric parameters are affected by the dose of dopaminergic medication such as L-DOPA, dopamine agonists, or MAO-B-I. Different types of dopaminergic treatments vary in their mechanisms of action, speed of action onset, and rate of plasma concentration decrease of the active substance^15^. Furthermore, the effect of dopaminergic medication may theoretically interact with autonomic dysfunction in PD. Therefore, it is not clear whether pupillometry is applicable in real-life scenarios and whether there are differences between the effect of L-DOPA and dopamine agonists on pupillary parameters.

The aim of this study was to evaluate the effect of different types of dopaminergic pharmacotherapy on pupillometric parameters in early-stage PD patients, examined in a treatment-naïve condition and then after one year of treatment in the practically defined OFF-medication and ON-medication states.

## Results

### Baseline clinical parameters and dopaminergic therapy initiation

A total of 63 patients (39 males, 24 females) completed the study and were included in the evaluation. The mean ± SD (range) age was 60.3 ± 11.5 (37–83), and the mean disease duration was 1.9 ± 1.8 (0.4–10.4) years, respectively. At baseline, the mean ± SD (range) MDS-UPDRS III score was 29.2 ± 10.9 (10–60). At the follow-up visit, 27 patients were treated with dopamine agonists, 24 patients by L-DOPA, 7 received a combined therapy of dopamine agonist and L-DOPA, and 5 were on dopamine agonist with MAO-I-B. The mean ± SD (range) L-DOPA equivalent daily dose (LEDD) was 454.9 ± 175.5 (160–1070) mg/day. The subgroup treated with L-DOPA monotherapy was older compared to subgroups on other medications (*p* < 0.001), while the subgroup treated with a combination of L-DOPA and dopamine agonist had the highest LEDD among all subgroups at the follow-up visit (*p* < 0.001). All patients in the dopamine agonist group used controlled release ropinirole in a single dose and their morning dose at the retest thus equaled the total daily dose. The mean morning dose at the retest in the L-DOPA group was 188 (SD 52, range 150–375) mg. Demographic and clinical characteristics of the PD patients divided according to the type of pharmacotherapy are listed in Table 1.

**Table 1:** Demographic and clinical characteristics of patients by type of pharmacotherapy.

	Dopamine agonist (*n* = 27)	L –DOPA (*n* = 24)	Dopamine agonist + L -DOPA (*n* = 7)	Dopamine agonist + IMAO (*n* = 5)	*p* – value	Post-hoc tests
**Baseline**						
Age (years)	53.6 ± 9.6	70.0 ± 7.1	55.7 ± 10.0	56.0 ± 8.1	**< 0.001**	LD>>> DA; LD > > LD + DA; LD > DA + IMAO
Male/female (n)	18/9	13/11	4/3	4/1	0.653	
Disease duration (years)	1.7 ± 1.4	2.0 ± 2.3	2.4 ± 1.9	1.4 ± 0.9	0.265	
MDS-UPDRS III†	27.9 ± 11.3 (29.0)	32.5 ± 10.1 (33.5)	30.5 ± 9.7 (34.0)	21.8 ± 6.5 (24.0)	0.104	
MoCA	26.0 ± 2.8	24.4 ± 432.0	24.7 ± 3.5	27.4 ± 1.9	0.847	
BDI-II	7.0 ± 3.7	10.9 ± 6.2	11.8 ± 7.2	8.4 ± 3.5	0.825	
STAI X1	40.0 ± 6.0	43.2 ± 10.9	41.2 ± 13.1	35.6 ± 5.2	0.419	
STAI X2	39.8 ± 7.6	45.0 ± 10.0	42.2 ± 10.6	33.0 ± 8.0	0.056	
**Retest OFF**						
MDS-UPDRS III†	26.3 ± 10.8 (26.5)	28.5 ± 10.3 (27.5)	25.9 ± 7.7 (25.5)	16.8 ± 2.7 (16.0)	0.131	
**Retest ON**						
MDS-UPDRS III†	22.7 ± 8.6 (20.0)	24.2 ± 9.9 (21.0)	22.9 ± 9.5 (29.0)	16.2 ± 8.7 (13.0)	0.350	
STAI X1†	37.3 ± 8.5 (36.0)	38.2 ± 8.1 (38.0)	38.4 ± 8.3 (33.0)	34.4 ± 8.6 (31.0)	0.864	
STAI X2†	38.5 ± 7.9 (38.0)	43.6 ± 7.2 (41.5)	39.5 ± 7.2 (35.0)	32.4 ± 8.6 (30.0)	0.068	
LEDD (mg)	343.0 ± 51.8	517.7 ± 93.7	775.7 ± 217.6	338.0 ± 29.9	**< 0.001**	LD>>> DA; LD<<< LD + DA; LD > > DA + IMAO; DA<<< LD + DA; LD + DA>>> DA + IMAO

Male/female distribution was compared using Chi-squared test.

† Values are shown as mean ± SD (median); analysis performed using Kruskal-Wallis test.

Other values are shown as mean ± SD; analysis performed using ANOVA.

Pairwise differences between treatment groups were assessed using Tukey’s HSD post-hoc test. Statistically significant differences are indicated as follows: (> or < ) *p* < 0.05, ( > > or << ) *p* < 0.01, (>>> or <<< ) *p* < 0.001.

LD = L-DOPA; DA = dopaminergic agonist; IMAO = monoaminooxidase B inhibitor; MDS-UPDRS III = Movement Disorder Society - Unified Parkinson’s Disease Rating Scale; MoCA = Montreal Cognitive Assessment; BDI = Beck Depression Inventory; STAI X1 = The State-Trait Anxiety Inventory X1; STAI X2 = The State-Trait Anxiety Inventory X2; LEDD = levodopa equivalent daily dose.

### Effect of dopaminergic therapy on pupillometric parameters and clinical characteristics

In the entire patient group, we observed a statistically significant decrease in the MDS-UPDRS III score from baseline to retest OFF (*p* = 0.004) and to retest ON (*p* < 0.001) and from retest OFF to retest ON (*p* < 0.001) conditions. Additionally, there was a significant decrease in the STAI X1 score from baseline to retest ON condition (*p* = 0.001). In the pupillometric analysis, significant effect of condition was detected for MAX, MIN, and VAR parameters. MAX and MIN parameters increased from baseline to retest ON (both *p* < 0.001) and from retest OFF to retest ON (*p* = 0.01 and *p* = 0.004, respectively). VAR parameter decreased from baseline to retest OFF and to retest ON (both *p* = 0.004); see Table 2.

**Table 2:** Pupillometric parameters in PD patients at baseline, retest OFF and retest ON.

	Baseline (*n* = 63)	Retest OFF (*n* = 63)	Retest ON (*n* = 63)	*p* - value	Post-hoc tests
MAX (mm)	4.4 ± 0.7 (4.4)	4.6 ± 0.8 (4.6)	4.8 ± 0.9 (4.8)	**< 0.001**	ON>>> baseline ON > OFF
VAR (%)	41.7 ± 6.8 (42.0)	40.3 ± 9.6 (39.0)	39.5 ± 6.5 (39.0)	**< 0.001**	ON < < baseline OFF < < baseline
DIF (mm)	1.9 ± 0.5 (1.8)	1.9 ± 0.5 (1.8)	1.9 ± 0.5 (1.9)	0.609	
MIN (mm)	2.5 ± 0.4 (2.5)	2.7 ± 0.7 (2.7)	2.9 ± 0.6 (2.8)	**< 0.001**	ON>>> baseline ON > > OFF
VC_max_ (mm/s)	5.0 ± 1.9 (4.6)	5.1 ± 2.6 (4.7)	5.5 ± 3.0 (4.9)	0.739	
T (ms)	205.4 ± 61.8 (214.0)	219.3 ± 48.9 (229.0)	209.8 ± 61.3 (222.0)	0.153	
MDS-UPDRS III	29.9 ± 11.5 (30.5)	26.7 ± 10.1 (26.5)	22.8 ± 9.3 (20.0)	**< 0.001**	ON<<< baseline; OFF < < baseline; ON<<< OFF
STAI X1	41.5 ± 9.6 (40.0)	n.d.	37.0 ± 8.4 (36.0)	**0.001**	
STAI X2	41.5 ± 9.4 (43.0)	n.d.	39.8 ± 8.4 (39.0)	0.065	

Values are shown as mean ± SD (median); analysis performed using Friedman or Wilcoxon signed-rank test.

Pairwise differences between visits were assessed using Wilcoxon post-hoc test with Holm-Bonferroni correction for multiple comparisons. Statistically significant differences are indicated as follows: (> or < ) *p* < 0.05, ( > > or << ) *p* < 0.01, (>>> or <<< ) *p* < 0.001.

ON = retest ON; OFF = retest OFF; n.d. = not done; MAX = maximum pupil diameter; VAR = variation ((MAX – MIN) ÷ MAX) × 100; DIF = difference between MAX and MIN; MIN = minimum pupil diameter; VC_max_ = maximum constriction velocity; T = latency for the onset of constriction; MDS-UPDRS III = Movement Disorder Society - Unified Parkinson’s Disease Rating Scale; STAI X1 = The State-Trait Anxiety Inventory X1; STAI X2 = The State-Trait Anxiety Inventory X2.

A linear mixed-effects analysis in subgroups receiving L-DOPA and dopamine agonist pharmacotherapy confirmed a significant effect of condition on the pupillometric parameters MAX (*p* < 0.001) and MIN (*p* = 0.005) while it did not show significant effect of treatment type for MAX (*p* = 0.106) or MIN (*p* = 0.102) parameters or interaction between condition and treatment type (*p* = 0.549 and *p* = 0.837 for MAX and MIN parameters, respectively).

**Fig. 1:**
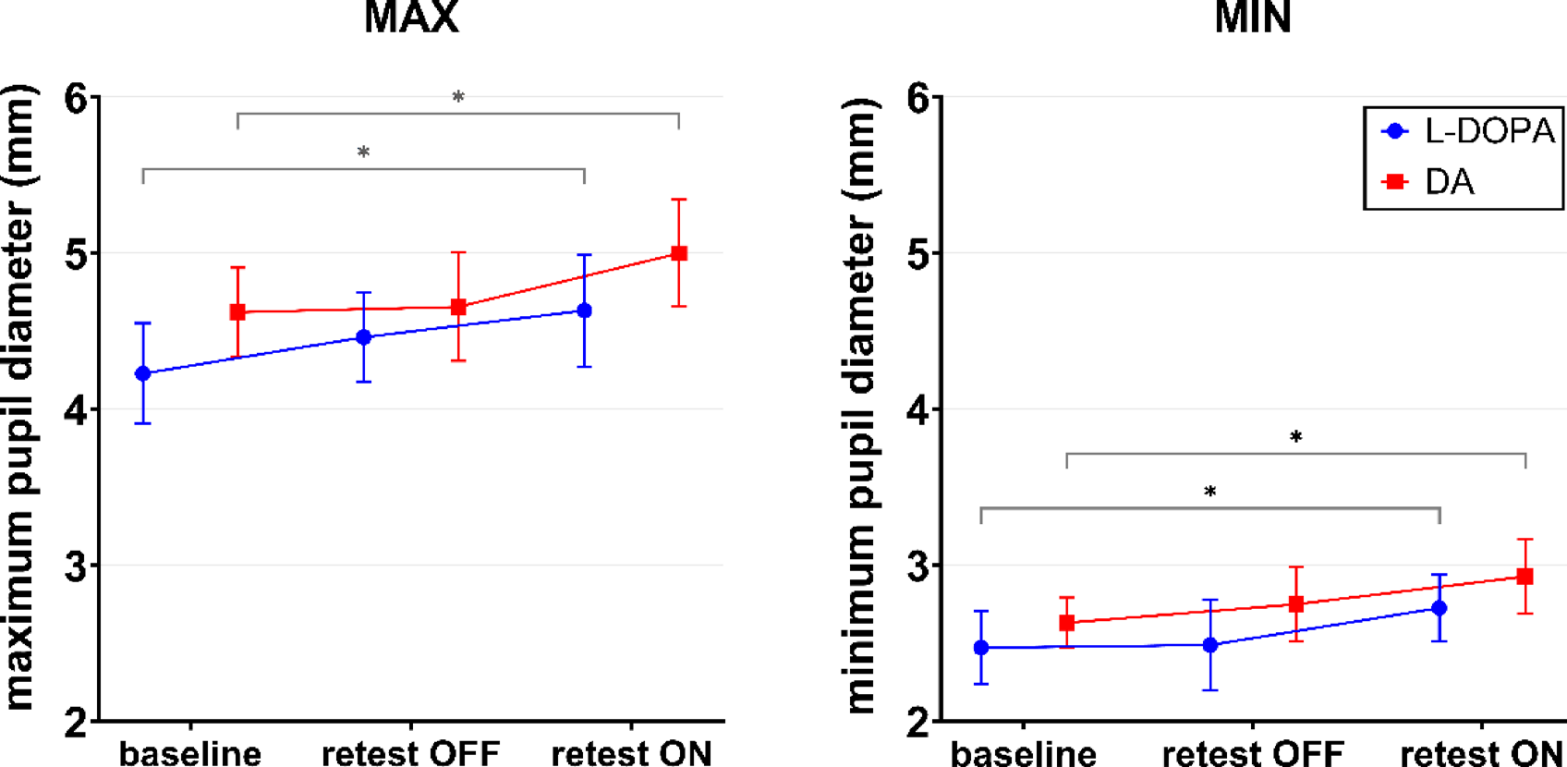
Effect of condition by treatment group for maximum and minimum pupil diameter. Symbols and error bars represent means and 95% confidence intervals. Statistically significant main effects of the linear mixed-effects model with subsequent Tukey’s multiple comparison test are shown. * adjusted p-value < 0.05. DA = dopamine agonist.

Exploring group-specific effects revealed a significantly larger MAX parameter at retest ON compared to baseline in both the dopamine agonist (*p* = 0.024) and L-DOPA (*p* = 0.016) subgroups as well as a significantly larger MIN parameter at retest ON compared to baseline in the dopamine agonist (*p* = 0.040) and L-DOPA (*p* = 0.028) subgroups (Fig. 1). Due to violation of the assumption of normality of residuals (Shapiro–Wilk *p* < 0.01), results were complemented by a non-parametric permutation-based ANOVA, which confirmed the main findings, significant increase in MAX and MIN parameters from baseline to retest ON condition in dopamine agonist (*p* = 0.037 and *p* = 0.008, respectively) and L-DOPA (*p* = 0.029 and *p* = 0.022, respectively) subgroups.

In both groups, there was a significant reduction in MDS-UPDRS III scores between baseline and retest ON as well as between retest OFF and retest ON (*p* < 0.001 for all effects; Supplementary Fig. S1). There were no significant differences in MDS-UPDRS III scores between the L-DOPA and agonist subgroups (Table 3).

**Table 3:** Pupillometric parameters in PD patients with dopamine agonist and with L – DOPA at baseline, retest OFF and retest ON.

PD patients with dopamine agonist				
	baseline (*n* = 27)	Retest OFF (*n* = 27)	retest ON (*n* = 27)	Significant effects (*p*-value)
MAX (mm)	4.6 ± 0.7	4.7 ± 0.9	5.0 ± 0.9	Baseline vs. ON (*p* = 0.024)
VAR (%)	42.6 ± 6.8	41.2 ± 12.3	39.9 ± 8.7	n.s.
DIF (mm)	2.0 ± 0.5	1.9 ± 0.6	2.0 ± 0.6	n.s.
MIN (mm)	2.6 ± 0.4	2.8 ± 0.7	2.9 ± 0.6	Baseline vs. ON (*p* = 0.040)
VC_max_ (mm/s)	5.3 ± 1.8	5.1 ± 2.3	6.4 ± 3.9	n.s.
T (ms)	201.0 ± 53.3	213.1 ± 43.5	200.2 ± 63.8	n.s.
MDS-UPDRS III	27.9 ± 11.3	26.3 ± 10.8	22.7 ± 8.6	Baseline vs. ON (*p* < 0.001); OFF vs. ON (*p* < 0.001)
**PD patients with L – DOPA**				
	Baseline (*n* = 24)	Retest OFF (*n* = 24)	Retest ON (*n* = 24)	
MAX (mm)	4.2 ± 0.8	4.5 ± 0.7	4.6 ± 0.8	Baseline vs. ON (*p* = 0.016)
VAR (%)	41.7 ± 7.4	42.3 ± 11.0	39.7 ± 4.2	n.s.
DIF (mm)	1.8 ± 0.4	1.9 ± 0.5	1.9 ± 0.4	n.s.
MIN (mm)	2.5 ± 0.5	2.5 ± 0.7	2.7 ± 0.5	Baseline vs. ON (*p* = 0.028)
VC_max_ (mm/s)	5.0 ± 2.3	6.0 ± 3.3	5.0 ± 2.0	n.s.
T (ms)	205.1 ± 76.6	220.8 ± 58.8	211.6 ± 62.2	n.s.
MDS-UPDRS III	32.5 ± 10.1	28.5 ± 10.3	24.2 ± 9.9	Baseline vs. ON (*p* < 0.001); OFF vs. ON (*p* < 0.001)

Values are shown as mean ± SD.

Analysis performed using linear mixed-efects model with Geisser-Greenhouse correction and subsequent Tukey’s multiple comparison test. Statistically significant main effects with adjusted p-values are shown.

ON = retest ON; OFF = retest OFF; MAX = maximum pupil diameter; VAR = variation ((MAX – MIN) ÷ MAX) × 100; DIF = difference between MAX and MIN; MIN = minimum pupil diameter; VC_max_ = maximum constriction velocity; T = latency for the onset of constriction; MDS-UPDRS III = Movement Disorder Society - Unified Parkinson’s Disease Rating Scale; n.s. = not significant.

An analysis of the pupillometric parameters in the subgroups of patients receiving a combination of L-DOPA and dopamine agonist as well as those receiving a combination of dopamine agonist and MAO-I-B is provided in Supplementary Table S1.

The mean increase between baseline and retest ON in the subgroups treated with L-DOPA and dopamine agonist was 0.4 ± 0.7 mm and 0.4 ± 0.7 mm, respectively, for the MAX parameter (Mann-Whitney U test, *p* = 0.94), and 0.3 ± 0.5 mm and 0.3 ± 0.6 mm, respectively for the MIN parameter (*p* = 0.56).

Spearman correlation analysis in the entire patient group found no significant associations between MDS-UPDRS III, STAI X1/2 scores, and pupillometric parameters in any condition (Supplementary Table S2). Similarly, no significant correlations were found between the differences in MDS-UPDRS III scores from baseline to retest ON, or from retest ON to retest OFF, and the corresponding differences in pupillometric parameters (data not shown).

### Sensitivity analyses taking age into account

Given the significant age difference between patient groups taking L-DOPA and dopamine agonist and the known effect of age on several pupillometric parameters, we performed several sensitivity analyses. Regression analysis between age and pupillometric parameters at baseline shows a significant negative effect of age on the MAX (β= -0.02, *p* = 0.01) and DIF (β= -0.01, *p* = 0.007) parameters whereby the maximum pupillary diameter MAX decreases by 0.02 mm every year (Supplementary Fig. S2).

Thus, linear mixed-effects model was reanalyzed for MAX and DIF parameters after their adjustment for age. For the age-adjusted DIF parameter there was no significant effect of factors condition and treatment type. For the age-adjusted maximum pupillary diameter (MAX) parameter significant effect of condition was observed (*p* < 0.001); exploring group-specific effects showed significantly larger MAX parameter in retest ON condition compared to baseline for dopamine agonist (*p* = 0.017) and L-DOPA (*p* = 0.009) subgroups, confirming the results of age-unadjusted analysis (Supplementary Fig. S3).

### Dose response relationship

Age-adjusted linear regression analysis of the relationship between pupillometric parameters in ON state and morning L-DOPA equivalent dose showed a significant positive dose–response relationship for the MAX, MIN, and DIF parameters in both treatment groups taking L-DOPA (β = 0.013, *p* < 0.001; β = 0.007, *p* < 0.001; β = 0.006, *p* < 0.001, respectively) and dopamine agonist (β = 0.012, *p* < 0.001; β = 0.006, *p* < 0.001; β = 0.007, *p* = 0.001, respectively). Differences in LED slopes between treatment groups were tested using F-tests for the LED × group interaction term in a combined model. No significant differences in LED-related slopes were observed between the two treatment groups for any of the pupillary parameters (Supplementary Fig S4, Table S3).

## Discussion

Our study shows that the use of chronic dopaminergic medication leads to an increase in both the maximum and minimum pupillary diameter; this effect partially persists even after the clinically defined time of discontinuation of the medication (OFF state). The change in pupil size is dependent on the dose of dopaminergic medication converted to L-DOPA equivalent regardless the type of dopaminergic medication but it is not associated with clinical improvement of PD motor symptoms.

Previous studies in PD patients describe a correlation between the results of pupillometric measurements and the detection of subclinical autonomic dysfunction, disease duration, and dopaminergic dysfunction^14,16^. In our previous study it was shown that L-DOPA dosage correlates with pupillometric parameters in PD and may be used as a tool for monitoring of its effect^17^. In the current study, we add the knowledge that dopamine agonist and L-DOPA affect pupillary parameters identically after conversion to L-DOPA equivalent dose. Values obtained during the clinically defined medication withdrawal period (OFF state) were intermediate between baseline and the ON-medication state (ON state). These findings confirm that the effects of dopaminergic medication are not only related to its actual plasmatic concentration but can persist even after its short-term discontinuation. This is consistent with the known persistence of dopaminergic effects on motor symptoms, which can last for weeks after treatment withdrawal^18^, and suggests that long-term dopaminergic therapy may alter central autonomic tone, leading to changes in pupil dynamics.

Several studies described relationship between pupillometric parameters and autonomic dysfunction in PD^19–21^. Notably, pupillometry abnormalities were helpful in detecting subclinical autonomic dysfunction^14,16^. Other publication demonstrated correlation between pupillometry parameters and motor severity assessed by MDS-UPDRS III score^12,22^. A recent review concludes that pupillometry offers the potential for an objective biomarker for early diagnosis and disease monitoring in PD^23^. In our study, there was no significant correlation between pupillometric parameters and MDS-UPDRS III in any condition, nor between the change in MDS-UPDRS III and the change in pupillometric parameters indicating differential dopaminergic effects on pupillomotor and somatomotor circuits.

Decrease in pupil size and alterations in pupillary reflex with increasing age are well described^24–26^. Maximum pupil diameter and difference between maximum and minimum pupil diameter showed a significant dependence on age also in our study. The baseline pupil size was numerically larger in the dopamine agonist subgroup and this difference persisted also at the retest. However, age of PD patients taking dopamine agonist was on average 16.4 years lower compared to those taking L-DOPA. After adjusting for age, the size difference was no longer apparent, indicating that this difference was likely caused by age-related decrease in pupil size (Supplementary Fig. S2, Fig. S3). L-DOPA equivalent doses were lower and the duration of the disease was shorter in the dopamine agonist group. This is related to the influence of younger age in patients with dopamine agonist, who had relatively milder motor symptoms at baseline and required lower medication dose to achieve similar level of functioning as compared to the L-DOPA subgroup. Yet, relationship between pupillometric parameters and L-DOPA equivalent doses in both, L-DOPA and dopamine agonist groups showed a similar dose–response relationship suggesting that pupillary parameters may be considered as a proxy for the net dopaminergic effect in the CNS. The regression slope was steeper for the MAX compared to MIN parameter, leading to increasing MAX-MIN difference with increasing L-DOPA equivalent dose. Thus, quantitative pupillary reflex assessment, particularly maximal and minimal pupil diameters and their difference may be thus a universal clinical indicator in monitoring the dose/effect of dopaminergic medication.

Several study limitations should be noted. First, we did not measure blood concentrations of dopaminergic medications. While we assume that the administered dose correlates with blood levels, individual differences in absorption may challenge this assumption, potentially biasing the analysis of the medication’s effect on pupillometry. Additionally, patients were retested after one year in accordance with the BIO-PD study protocol. During this period, both the effects of treatment and the progression of neurodegenerative changes may have contributed to the differences observed between baseline and retest conditions. Also, autonomic dysfunction which is known to affect pupillary reactions and is variably present in early PD was not considered in this study and might have biased measurement in some of patients. We also did not consider dietary modifiers of pupillary function such as coffee or tea. Nevertheless, despite these limitations, the significant differences observed between the OFF and ON conditions clearly indicate an effect of the acute dose of dopaminergic medication. Lastly, the relatively small sample size limited our ability to analyze the effects of MAO-B inhibitors and other less commonly used antiparkinsonian medications.

## Conclusion

The use of chronic dopaminergic medication leads to an increase in both the maximum pupillary diameter and minimum pupillary diameter; this effect partially persists even after the clinically defined time of discontinuation of the medication. The change in pupil size is dependent on the dose of dopaminergic medication converted to L-DOPA equivalent, while there is no difference between the effect of dopamine agonist and L-DOPA. Pupillometry is a potentially useful tool for monitoring the effect of dopaminergic medication. Future studies should focus on the importance of pupillometry, pharmacogenetics and pharmacokinetics for the diagnosis, treatment and prognosis of PD patients.

## Methods

### Study participants

This prospective and low-intervention study was approved by the local Ethics Committee of the General University Hospital, Prague, Czech Republic under the number 17/19. This study has been conducted in accordance with the Declaration of Helsinki. Relevant Standard Operating Procedures for pupillometry measurements and clinical trials conduct were followed. All participants signed the informed consent.

63 PD patients consecutively enrolled into the longitudinal study BIO-PD^27^ from October 2018 to August 2023 were examined at the Department of Neurology, First Faculty of Medicine, Charles University and General University Hospital in Prague. BIO-PD is a longitudinal study designed to examine biomarkers of progression in *de novo* PD patients, with a protocol that includes annual follow-up visits.

All patients had *de novo*, untreated PD diagnosed according to the Movement Disorder Society (MDS) clinical diagnostic criteria^28^. Notably, the diagnosis was confirmed by abnormal dopamine transporter single photon emission computed tomography (DAT-SPECT) finding and alternative diagnosis (e.g. atypical or secondary parkinsonism) was excluded by clinical examination and brain magnetic resonance imaging (MRI). PD patients were free of any comorbid neurological disorder, eye disorder or any other diseases or conditions which could affect the pupillary reflex. In particular, no patient had diabetes mellitus, ocular surgery in the past, glaucoma under treatment, pseudo-exfoliation syndrome on the pupil margin or the lens, and corneal disease. No patient was taking medications containing cholinergic and adrenergic agents.

### Study protocol and methods

All patients were investigated at baseline, before the first application of dopaminergic therapy (baseline), and then at follow-up visit after one year of treatment. At the follow-up visit patients were first examined in the morning in a practically defined OFF-medication state (retest OFF; assessed after 12 h of levodopa and/or 48 h of dopamine agonist withdrawal) and then retested in the defined ON-medication state (retest ON; assessed approximately 60 min after the administration of the patient’s usual morning dose of levodopa and/or dopamine agonists). The 60-minute interval was chosen because the average time to reach maximum plasma concentration of levodopa is approximately 60 min. This interval was also applied to other medications to enable a standardized comparison across different pharmacotherapeutic regimens^29^. At baseline, retest OFF and retest ON, patients were assessed using pupillometry and the Movement Disorders Society-Unified Parkinson´s Disease Rating Scale (MDS-UPDRS) part III^30^. At baseline, the protocol additionally included Beck depression inventory (BDI-II) and Montreal cognitive assessment (MoCA)^31,32^. State-Trait Anxiety Inventory (STAI-X1/2), which captures situational anxiety (STAI X1) and general anxiety tendencies (STAI X2), was examined at baseline and retest ON conditions.

### Treatment

After the baseline examination, dopaminergic therapy was initiated. The choice of therapy was tailored on a patient-per-patient basis according to current guidelines. Patients with older age or internal co-morbidities were started on L-DOPA, DCI / L-DOPA with ratio of 1:4; in young patients, dopamine agonist was considered based on patients’ preference. The medication dose was titrated until adequate symptoms relief was reported by patients. In those with insufficient effect of dopamine agonists including those who did not tolerate desirable dopamine agonist doses due to adverse effects, adjunctive L-DOPA or MAO-B-I therapy was added. The daily doses of dopaminergic medication at the follow-up examination were recalculated to L-DOPA equivalent daily dose (LEDD)^33^.

### Pupillometry

Pupillometry was performed by a single examiner using a monocular infrared pupillograph, the Compact Video Pupillometer NeuroLight (IDMed, Marseille, France). The measurements were conducted on the right eye of each subject in a quiet and fully darkened room after at least 5 min of dark adaptation. Subjects were instructed to look straight at the red fixation point. Stimulation involved a 320 lx light flash for 1 s, followed by a 4-second recording period. The system captured images at a rate of 67 images per second. Static pupillometry consisted of measurement of maximum pupil diameter (MAX), whereas dynamic parameters included minimum pupil diameter (MIN), difference between MAX and MIN (DIF), variation ((MAX – MIN) ÷ MAX) × 100 (VAR), maximum constriction velocity (VC_max_) and latency for the onset of constriction (T)^34^. Each value was calculated as a median of the first five artefact-free measurements. The time interval between individual measurements was approximately three seconds.

### Statistical analysis

The normality of distributions was assessed using the Shapiro-Wilk test. Differences in baseline demographics and clinical characteristics of patients with different types of pharmacotherapy were tested with one-way ANOVA for normally distributed data and Kruskal-Wallis test for non-normally distributed data. Categorical variables were analyzed using the Chi-squared test.

Longitudinal within-subject differences in clinical and pupillometric parameters were tested using one-way ANOVA with repeated measures, Friedman or Wilcoxon signed-rank test as appropriate. The relationships between pupillometric and clinical variables were analyzed via Spearman’s correlation coefficient.

Both, treatment type (L-DOPA vs. dopamine agonist) and condition (baseline vs. retest OFF vs. retest ON conditions) factors were tested with a linear mixed-effects model with subsequent Tukey´s multiple comparisons test. Full models for each pupillometric parameter included fixed effects for condition, group, and their interaction. Linear multivariable regression analysis was used for testing the relationship between pupillometry parameters and morning L-DOPA equivalent doses in both subgroups taking either L-DOPA or dopaminergic agonist adjusted for age. Regression slopes between L-DOPA and dopaminergic agonist were compared using the F-test for LED by group interaction. Homoscedasticity and normality of model residuals were evaluated visually using residuals versus a fitted plot and a quantile-quantile plot and were also verified with the Levene and Shapiro-Wilk tests, respectively. Confirmatory analysis using non-parametric permutation-based ANOVA (1000 iterations) and post-hoc Wilcoxon signed-rank tests with Holm correction were performed when assumptions of linear regression were not met.

Holm-Bonferroni correction was used to control the family-wise error rate in multiple hypothesis testing. For sensitivity analysis, age-dependent pupillary parameters were adjusted for age using the regression coefficient (β) and centered at the mean age. Statistical analysis was performed using Python in a Jupyter-notebook environment and GraphPad Prism software version 8.2.1 (GraphPad Inc., La Jolla, CA, USA). P-levels < 0.05 were considered as statistically significant.

## Supplementary Information

Below is the link to the electronic supplementary material.

Supplementary Material 1

## Data Availability

Data are available on reasonable request from the corresponding authors.
