# Supplementary material for: Longitudinal analysis of pupillometry according to type of pharmacotherapy in Parkinson’s disease patients
Source: Sci Rep. 2025 Aug 20;15:30525. doi: 10.1038/s41598-025-15306-z (PMC12368211; doi:10.1038/s41598-025-15306-z)
Supplement: Supplementary file 1 — Supplementary Material 1 [file 41598_2025_15306_MOESM1_ESM.docx]

**Supplementary Table S1:** Pupillometric parameters in PD patients with the combination of L-DOPA and dopamine agonist, and with the combination of dopamine agonist and MAO-I-B in baseline, retest OFF and retest ON.

| **PD patients with the combination of L-DOPA and dopamine agonist** | | | | |
| --- | --- | --- | --- | --- |
|  | baseline  (n = 7) | Retest OFF  (n = 7) | retest ON  (n = 7) | P - value |
| MAX (mm) | 4.2 ± 0.4 (4.1) | 5.0 ± 1.1 (4.6) | 5.2 ± 0.8 (5.0) | 0.102 |
| VAR (%) | 40.4 ± 5.3 (41.0) | 38.4 ± 3.0 (39.0) | 39.5 ± 3.6 (28.0) | 0.179 |
| DIF (mm) | 1.7 ± 0.3 (1.7) | 1.9 ± 0.4 (1.8) | 2.1 ± 0.2 (2.1) | 0.368 |
| MIN (mm) | 2.5 ± 0.2 (2.4) | 3.0 ± 0.7 (3.1) | 3.1 ± 0.6 (3.1) | 0.156 |
| VC_max_ (mm/s) | 4.1 ± 0.8 (4.3) | 4.4 ± 0.8 (4.2) | 5.0 ± 0.9 (4.9) | 0.368 |
| T (ms) | 216.0 ± 16.0 (213.0) | 219.3 ± 28.2 (226.0) | 228.4 ± 17.5 (222.0) | 0.368 |
| MDS-UPDRS III | 30.9 ± 10.0 (34.0) | 25.2 ± 6.5 (25.5) | 22.7 ± 9.0 (29.0) | **0.016** |
| **PD patients with the combination of dopamine agonist and MAO-I-B** | | | | |
|  | baseline  (n = 5) | retest OFF  (n = 5) | retest ON  (n = 5) | P - value |
| MAX (mm) | 4.3 ± 0.8 (4.4) | 4.7 ± 0.9 (5.0) | 4.4 ± 0.8 (4.6) | 0.854 |
| VAR (%) | 38.8 ± 3.2 (40.0) | 37.8 ± 4.3 (38.0) | 36.2 ± 4.4 (39.0) | 0.241 |
| DIF (mm) | 1.8 ± 0.4 (1.9) | 1.8 ± 0.5 (1.9) | 1.6 ± 0.5 (1.9) | 0.819 |
| MIN (mm) | 2.6 ± 0.4 (2.6) | 2.8 ± 0.5 (2.9) | 2.8 ± 0.5 (2.8) | 0.819 |
| VC_max_ (mm/s) | 4.8 ± 1.9 (4.6) | 4.2 ± 1.2 (4.7) | 3.8 ± 1.2 (4.4) | 0.819 |
| T (ms) | 216.0 ± 62.1 (220.0) | 246.0 ± 36.6 (235.0) | 226.4 ± 71.0 (231.0) | 0.549 |
| MDS-UPDRS III | 21.8 ± 5.8 (24.0) | 16.8 ± 2.7 (16.0) | 16.2 ± 8.7 (13.0) | 0.109 |

Values are shown as mean ± SD (median); analysis performed using Friedman test

Abbreviations: MAX = maximum pupil diameter; VAR = variation ((MAX – MIN) ÷ MAX) × 100; DIF = difference between MAX and MIN; MIN = minimum pupil diameter; VC_max_ = maximum constriction velocity; T = latency for the onset of constriction; MDS-UPDRS III = Movement Disorder Society - Unified Parkinson's Disease Rating Scale; MAO-I-B = Monoamine oxidase-B inhibitors.

**Supplementary Table S2:** Correlation analysis of pupillary parameters and clinical variables at baseline, retest OFF and retest ON condition.

|  | **baseline** | | | **retest OFF** | **retest ON** | | |
| --- | --- | --- | --- | --- | --- | --- | --- |
|  | MDS-UPDRS III | STAI-X1 | STAI-X2 | MDS-UPDRS III | MDS-UPDRS III | STAI-X1 | STAI-X2 |
| **MAX** | 0.05 | 0.09 | 0.06 | -0.04 | 0.00 | 0.08 | 0.11 |
| **VAR** | 0.08 | 0.12 | 0.04 | -0.16 | -0.22 | 0.08 | 0.12 |
| **DIF** | 0.06 | 0.10 | 0.05 | -0.22 | -0.11 | 0.08 | 0.11 |
| **MIN** | 0.05 | 0.03 | 0.13 | 0.06 | 0.04 | 0.03 | 0.11 |
| **VC_max_** | 0.24 | 0.16 | 0.07 | -0.06 | -0.02 | 0.14 | 0.20 |
| **T** | -0.23 | 0.14 | 0.24 | -0.05 | -0.10 | -0.17 | -0.13 |

Spearman rho (r_s_) values for individual correlation analyses are shown.

Abbreviations: MAX = maximum pupil diameter; VAR = variation ((MAX – MIN) ÷ MAX) × 100; DIF = difference between MAX and MIN; MIN = minimum pupil diameter; VC_max_ = maximum constriction velocity; T = latency for the onset of constriction; MDS-UPDRS III = Movement Disorder Society - Unified Parkinson's Disease Rating Scale; STAI = The State-Trait Anxiety Inventory.

**Supplementary Table S3:** Multiple linear regression analysis of the relationship between the morning L-DOPA equivalent dose and pupillometric parameters and age in subgroups with dopamine agonist and L-DOPA in the retest ON condition.

|  | **Dopamine agonist (n=27)** | | | **L-DOPA (n=24)** | | | **Difference between slopes (LED x group)** |
| --- | --- | --- | --- | --- | --- | --- | --- |
|  | R^2^ | Slope LEDD (p-value) | Slope age (p-value) | R^2^ | Slope LEDD (p-value) | Slope age (p-value) |  |
| **MAX (mm)** | 0.502 | 0.012  **(p<0.001)** | -0.002 (p=0.917) | 0.666 | 0.013 **(p<0.001)** | -0.017 (p=0.266) | No (p=0.651) |
| **VAR (%)** | 0.083 | 0.038  (p=0.239) | 0.183 (p=0.315) | 0.038 | 0.017  (p=0.305) | -0.265 (p=0.038) | No (p=0.378) |
| **DIF (mm)** | 0.390 | 0.007  **(p=0.001)** | 0.013 (p=0.237) | 0.623 | 0.006 **(p<0.001)** | -0.020 (p=0.028) | No (p=0.606) |
| **MIN (mm)** | 0.490 | 0.006  **(p<0.001)** | 0.000 (p=0.995) | 0.549 | 0.007  **(p<0.001)** | 0.001 (p=0.933) | No (p=0.679) |
| **VC_max_ (mm/s)** | 0.253 | 0.035  (p=0.013) | 0.079 (p=0.294) | 0.163 | 0.003  (p=0.692) | -0.114 (p=0.068) | No (p=0.235) |
| **T (ms)** | 0.059 | 0.111  (p=0.686) | -1.447 (p=0.354) | 0.041 | 0.126  (p=0.645) | 1.558 (p=0.435) | No (p=0.361) |

Uncorrected p-values are reported; p-values significant after Holm-Bonferroni correction are shown in **BOLD** text. For multiplicity correction, analyses in both L-DOPA and dopaminergic agonist groups were treated as independent families of tests.

Differences in the slopes of the associations between pupillary parameters and LEDD across treatment groups were assessed using an F-test for the interaction term in a multiple linear regression model (LED × group).

Abbreviations: LED = L-DOPA equivalent dose; MAX = maximum pupil diameter; VAR = variation ((MAX – MIN) ÷ MAX) × 100; DIF = difference between MAX and MIN; MIN = minimum pupil diameter; VC_max_ = maximum constriction velocity; T = latency for the onset of constriction.

**Supplementary Fig. S1:** Effect of condition by treatment group for MDS-UPDRS III score.

**
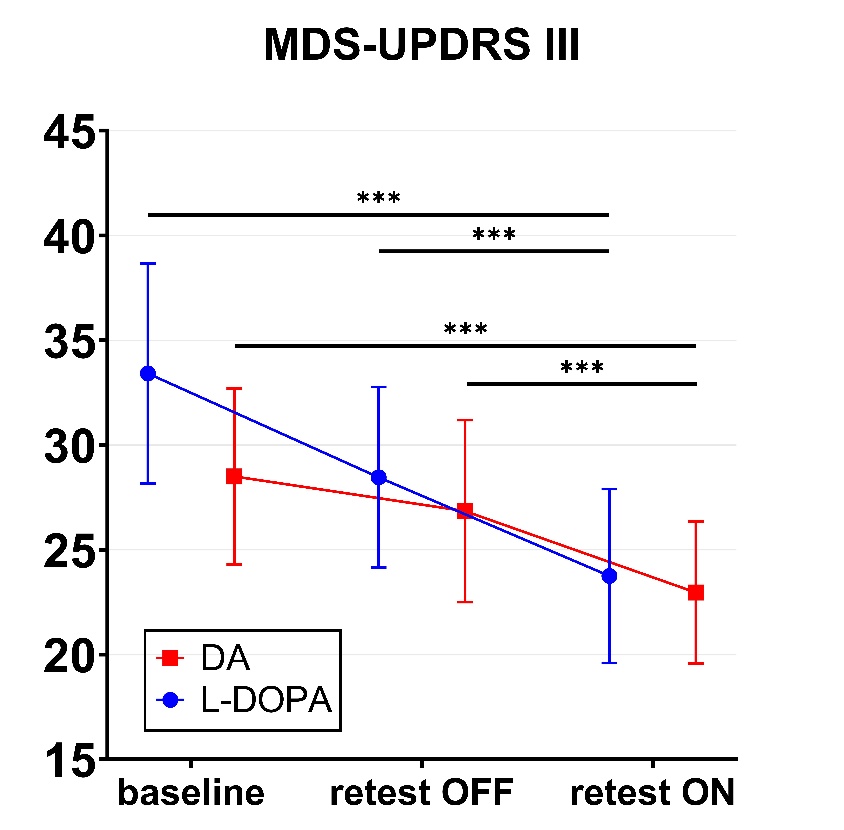
**

Symbols and error bars represent means and 95% confidence intervals. Statistically significant main effects of the linear mixed-effects model with subsequent Tukey’s multiple comparison test are shown. *** adjusted p-value <0.001

Abbreviations: DA = dopamine agonist; MDS-UPDRS III = Movement Disorder Society - Unified Parkinson's Disease Rating Scale.

**
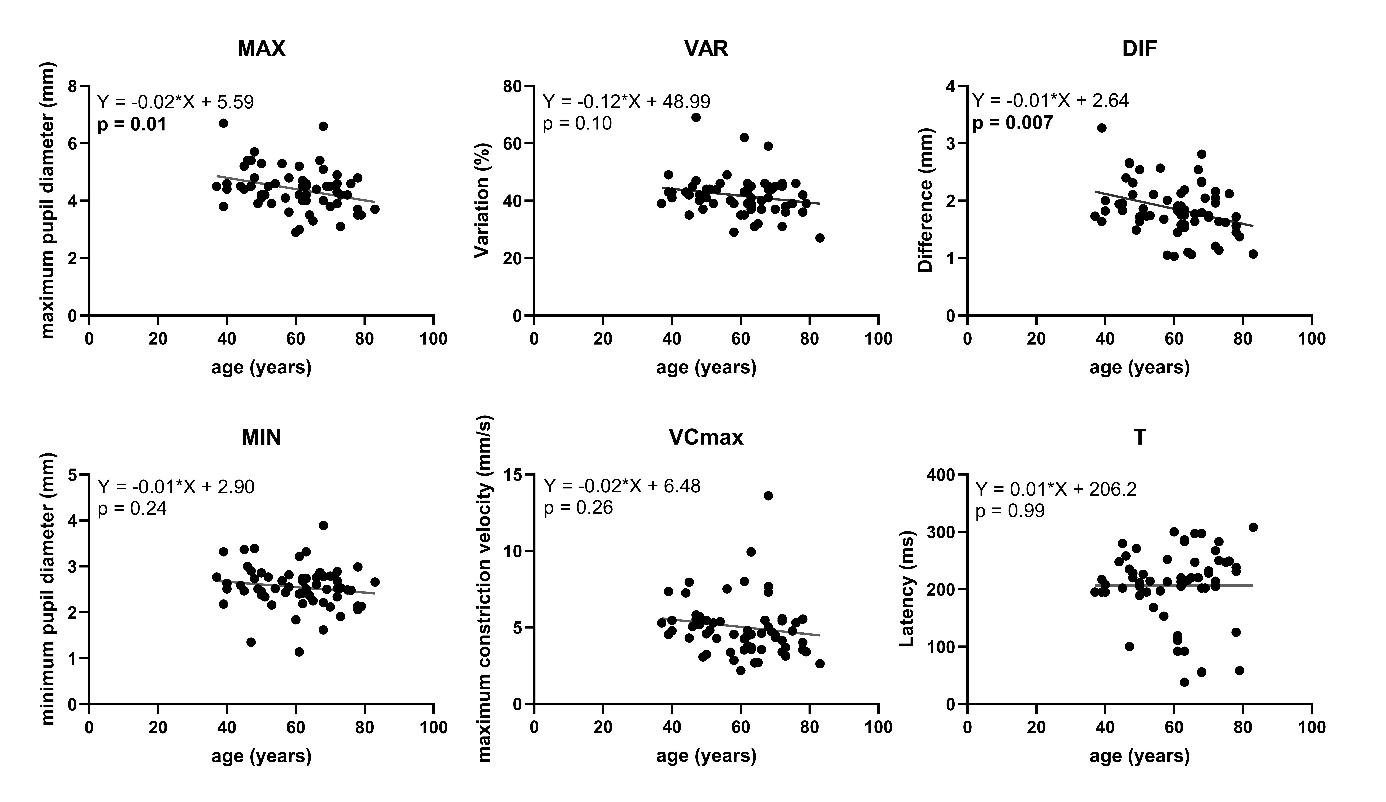
Supplementary Fig. S2:** The effect of age on pupillometric parameters.

Scatter plots with fitted regression lines and equations illustrate the linear relationships between age and each pupillometric parameter.

Abbreviations: MAX = maximum pupil diameter; VAR = variation ((MAX – MIN) ÷ MAX) × 100; DIF = difference between MAX and MIN; MIN = minimum pupil diameter; VC_max_ = maximum constriction velocity; T = latency for the onset of constriction.


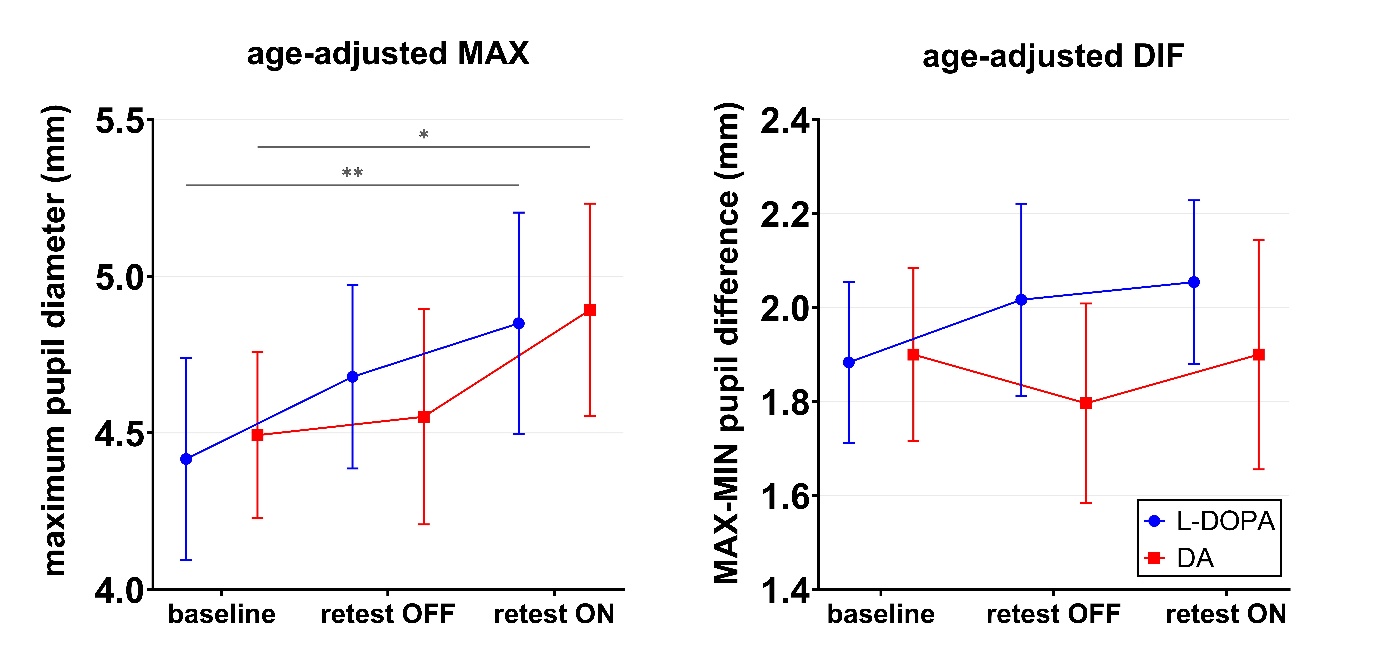
**Supplementary Fig. S3:** Effect of condition by treatment group for age-adjusted maximum pupil diameter and MAX-MIX pupil difference

Symbols and error bars represent means and 95% confidence intervals. Statistically significant main effects of the linear mixed-effects model with subsequent Tukey’s multiple comparison test are shown. * adjusted p-value <0.05; ** adjusted p-value <0.01. DA = dopamine agonist.


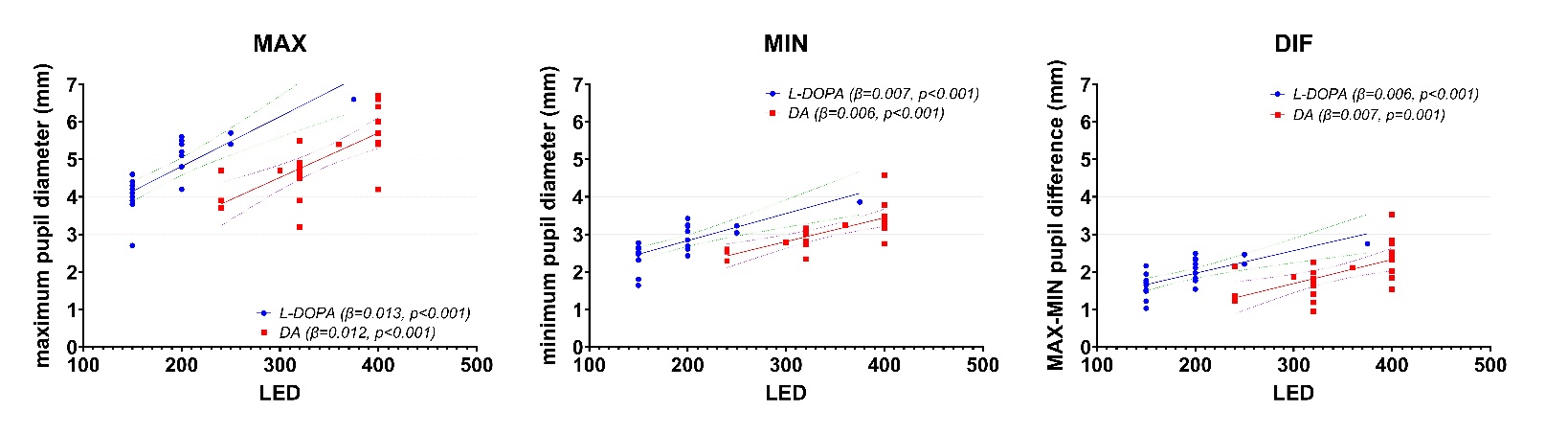
**Supplementary Fig. S4:** Relationship between pupillary parameters and morning L-DOPA equivalent dose at retest ON condition

Scatter plots with fitted regression lines and 95% confidence bands illustrate the linear relationships between morning LED and MAX, MIN, and DIF pupillometric parameter at retest ON condition.

Abbreviations: MAX = maximum pupil diameter; DIF = difference between MAX and MIN; MIN = minimum pupil diameter; LED = L-DOPA equivalent dose; DA = dopamine agonist.
